# Uncertain Inheritance: Transgenerational Effects of Environmental Exposures

**DOI:** 10.1289/ehp.121-A298

**Published:** 2013-10-01

**Authors:** Charles W. Schmidt

**Affiliations:** **Charles W. Schmidt**, MS, an award-winning science writer from Portland, ME, has written for *Discover Magazine*, *Science*, and *Nature Medicine*.

Andrea Cupp made a serendipitous discovery when she was a postdoctoral fellow at Washington State University: While investigating how chemicals affect sex determination in embryonic animals, she bred the offspring of pregnant rats that had been dosed with an insecticide called methoxyclor. When the males from that litter grew into adults, they had decreased sperm counts and higher rates of infertility. Cupp had seen these same abnormalities in the animals’ fathers, which had been exposed to methoxyclor in the womb. But this latest generation hadn’t been exposed that way, which suggested that methoxyclor’s toxic effects had carried over generations. “At first I couldn’t believe it,” says Cupp’s advisor, Michael Skinner, a biochemist and Washington State professor. “But then we repeated the breeding experiments and found that the results held up.”

Skinner and Cupp, who is now a professor at the University of Nebraska–Lincoln, published their findings in 2005.^^1^^ Since that paper—which showed that reproductive effects not just from methoxyclor but also from the fungicide vinclozolin persisted for at least four generations—the number of published articles reporting similar transgenerational findings has increased steadily. “In the last year and half there’s been an explosion in studies showing transgenerational effects from exposure to a wide array of environmental stressors,” says Lisa Chadwick, a program administrator at the National Institute of Environmental Health Sciences (NIEHS). “This is a field that’s really starting to take off.”

According to Chadwick, the new findings compel a reevaluation of how scientists perceive environmental health threats. “We have to think more long-term about the effects of chemicals that we’re exposed to every day,” she says. “This new research suggests they could have consequences not just for our own health and for that of our children, but also for the health of generations to come.”

**Figure 1:**
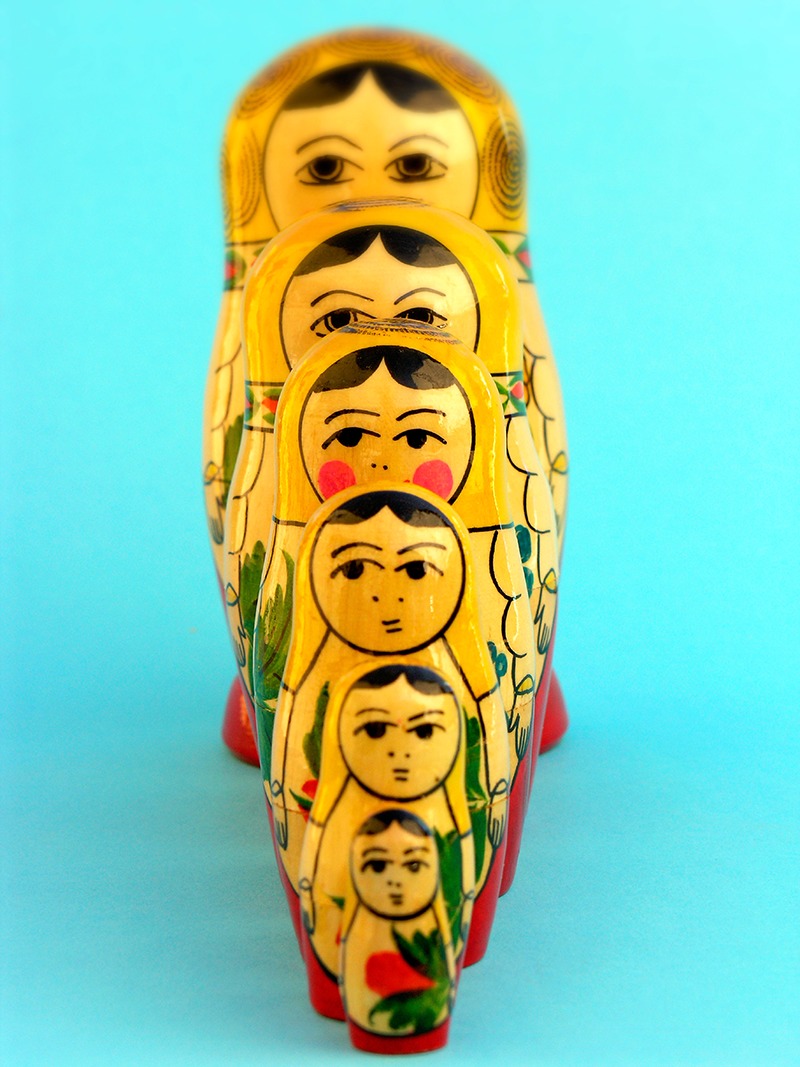
Glossary **Epigenetic**—Refers to alterations in gene expression potential that can be passed down through generations. **F_0_, F_1_, etc.**—Shorthand used to distinguish successive generations from one another. “F” stands for “filial generation.” **Germ line**—The genetic lineage of germ cells (egg and sperm progenitors) that passes down through generations of individuals. **Imprinted gene**—A gene whose expression is determined by whether it comes from the mother or the father. **Marks (or Tags)**—Molecules that attach to DNA and influence gene expression. **Methylation**—Modification of DNA by the addition of a type of molecule known as a methyl group. **Multigenerational**—Refers to effects that extend to the F_2_ (grandchild) generation. **NOAEL**—The highest dose that produces no adverse effects in exposed animals during a toxicology study. **Transgenerational**—Refers to effects that extend to the F_3_ (great-grandchild) generation.

The NIEHS recently issued requests for applications totaling $3 million for research on transgenerational effects in mammals.^^2^^ Chadwick says funded studies will address two fundamental data needs, one pertaining to potential transgenerational mechanisms and another to the number of chemicals thought to exert these effects. These studies will extend to what’s known as the F_3_ generation—the great-grandchildren of the originally exposed animal. That’s because chemicals given to pregnant females (the F_0_ generation) interact not only with the fetal offspring (the F_1_ generation) but also the germ cells developing within those offspring, which mature into the sperm and eggs that give rise to the F_2_ generation. Thus, the F_3_ animals are the first generation to be totally unexposed to the original agent. Effects that extend to the F_2_ generation are known as “multigenerational,” whereas those that extend to the F_3_ generation are known as “transgenerational.”^^3^^

Transgenerational effects have now been reported for chemicals including permethrin, DEET, bisphenol A, certain phthalates, dioxin, jet fuel mixtures, nicotine, and tributyltin, among others. Most of these findings come from rodent studies.^^4^^^,^^^5^^^,^^^6^^^,^^^7^^ But preliminary evidence that chemical effects can carry over generations in humans is also emerging, although no F_3_ data have been published yet. Given the challenges of tracking effects over multiple human lifespans, the evidence is more difficult to interpret, particularly with respect to potential mechanisms, says Tessa Roseboom, a professor of early development and health at the Academic Medical Center in Amsterdam, the Netherlands. Still, some reports have linked nutritional deficiencies from famine and exposure to diethylstilbestrol (DES)—a nonsteroidal estrogen used to protect against miscarriage from the 1940s to the 1970s—to effects that persist among the grandchildren of exposed women.^^8^^^,^^^9^^^,^^^10^^^,^^^11^^^,^^^12^^^,^^^13^^

## Foundations in Animal Data

The way in which environmental exposures cause transgenerational effects is unclear. According to Chadwick, current hypotheses lean toward epigenetic inheritance patterns, which involve chemical modifications to the DNA rather than mutations of the DNA sequence itself. Scientists debate the precise definition of “epigenetics,” but Robert Waterland, an associate professor of pediatrics and genetics at Baylor College of Medicine, suggests the best definition was published in *Nature Genetics* 10 years ago: “The study of stable alterations in gene expression potential that arise during development and cell proliferation.”^^14^^

Epigenetic modifications can take a few different forms—molecules known as methyl groups can attach to DNA itself, or methyl or acetyl groups can attach to the histone proteins that surround DNA. These attached molecules, also known as “marks” or “tags,” influence gene expression and thereby determine the specialized function of every cell in the organism.

Epigenetic marks carried over from the parents are typically wiped clean during molecular programming events that happen early in embryonic development. Shortly after fertilization, explains Dana Dolinoy, an assistant professor at the University of Michigan School of Public Health, a wave of DNA demethylation leaves the embryo with a fresh genomic slate with the exception of certain imprinted genes, such as insulin-like growth factor 2 (*IGF2*), which remain methylated. Later, cells in the developing embryo are remethylated as they develop into the somatic cells that make up different organs and tissues in the body. Germ cells, meanwhile, undergo their own wave of demethylation and remethylation programming events, which are specific to the sex of the developing embryo.

Researchers have found that transgenerational effects can result from chemical dosing at precise windows in fetal development—specifically, at the time of sex determination, which occurs around embryonic days 10.5–12.5 for mice and embryonic days 41–44 for humans, according to Duke University cell biology professor Blanche Capel. Observable effects in the F_3_ generation are thought to result from changes to the germ line, which is the succession of germ cell DNA that passes from one generation to the next. Skinner and other researchers have identified DNA methylation changes in F_3_-generation sperm that appear to underlie transgenerational effects seen in F_3_ animals.^^1^^^^,^^^^4^^

Researchers emphasize that much of the evidence so far in the field is observational, meaning the biological mechanisms remain unknown. Dolinoy says scientific opinions lean heavily toward epigenetic pathways. “That seems to be where the whole field is headed,” she says.

According to Chadwick, Skinner’s laboratory remains a nexus for transgenerational studies in chemically exposed animals. In his more recent work, Skinner has shown that insecticides, phthalates, dioxin, and jet fuel, when given to gestating rats during periods of embryonic programming, promote early-onset puberty in female offspring and decreased sperm counts in males, out to the F_3_ generation.^^4^^ “We mapped DNA methylation in germ cells and found that each compound induces a unique epigenetic signature,” Skinner says. “But it’s also possible that other epigenetic mechanisms play a role.”

Meanwhile, several other groups are studying transgenerational changes in animals. In one study, Kwan Hee Kim, a professor of molecular biosciences at Washington State, exposed pregnant mice to di-(2-ethylhexyl) phthalate (DEHP) on embryonic days 7–14.^^7^^ Kim observed decreased sperm counts and sperm motility in male offspring out to the F_4_ generation. Importantly, she also observed an 80% reduction in spermatogonial stem cell regeneration. Consequently, she says, “As the animals aged, their ability to make new sperm decreased dramatically.”

Kim implicates DNA methylation as a potential epigenetic mechanism behind the change in function. During the study, she identified 16 genes that were differentially methylated and expressed in newborn pups, she says. This group of targeted genes may hold clues to how DEHP acts transgenerationally.

In another new study, Virender Rehan, a professor of pediatrics at the Harbor–UCLA Medical Center, found that prenatal exposure to nicotine in rats starting at embryonic day 6 was associated with asthma-like symptoms among F_3_ males and females. But, similarly to an earlier study extending to F_2_ offspring, the effects were sex-specific, with total airway system resistance significantly greater in males than females, due in part to tracheal constriction, which was detected only in males.^^5^^^^,^^^^6^^ What’s still unclear (and a subject of his current research), Rehan says, is whether the transgenerational effect is being carried through the male or female germ line.

**Figure 2:**
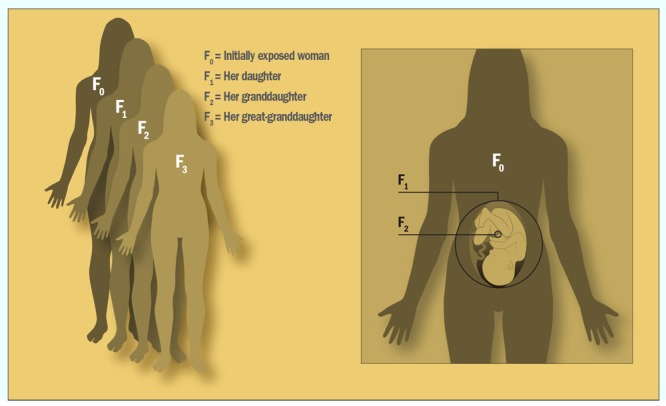
When a pregnant woman is exposed to an environmental agent, the exposure extends not only to herself (F_0_) and her unborn child (F_1_), but also to the germ cells developing within the fetus (F_2_). Animal studies have demonstrated chemical effects that extend a generation further still—to the F_3_ generation, the first generation not directly exposed to the original agent. Human studies to date have shown effects only through the F_2_ generation. Joseph Tart/EHP

Bruce Blumberg, a professor of developmental and cell biology at the University of California, Irvine, recently published a mouse study showing that maternal exposure to the biocide tributyltin (TBT) induced a condition similar to nonalcoholic fatty liver disease out to the F_3_ generation.^^15^^ Like other transgenerational toxicants, TBT is an endocrine disruptor that appears to be an obesogen, or a chemical that promotes obesity partly by promoting the growth of fat cells.^^16^^ Blumberg’s study used doses as much as 50-fold lower than the no observed adverse effect level (NOAEL) for TBT.

According to Blumberg, the findings also support an evolving concept in reproductive biology—the “developmental origins of health and disease” hypothesis, which holds that low-dose chemical exposures or maternal dietary changes experienced *in utero* can induce permanent physical changes in adult animals.^^17^^ “These effects are permanent in that they remain even when you take away the exposure,” he says. “Now we’re finding that the effects can also last through subsequent generations.”

Other researchers have found evidence that transgenerational effects can impact mating behaviors, with implications for the evolution of populations. In one example, David Crews, a professor of biology and psychology at the University of Texas at Austin, reported that female rats avoided F_3_ males with an ancestral exposure to vinclozolin. The study specifically found that all females tested preferred control males (who had no ancestral vinclozolin exposure) whereas males from both the control and ancestrally treated groups exhibited no particular preference for female type.^^18^^ “Where the rubber meets the road in evolution is sex,” Crews says. “It’s all about who mates and reproduces with who.”

## The Case for Multigenerational Effects in Humans

The evidence for environmentally induced multigenerational effects in humans began to emerge years ago from an isolated community in Northern Sweden called Överkalix Parish. Led in part by Marcus Pembrey, a clinical geneticist at the University College London Institute of Child Health, researchers investigated whether an abundance of food in childhood had any influence on the risk of heart disease and diabetes among a child’s future descendants. In particular, the researchers studied overeating during a child’s “slow-growth period,” the lull before the prepubertal growth spurt.

An initial study published in 2002 suggested the answer was a conditional yes. By studying harvest statistics, grain prices, and other records, the researchers classified food availability in Överkalix for individual years of the nineteenth century as poor, moderate, or good. They then studied health outcomes among descendants born in 1890, 1905, and 1920, and found that food abundance during the grandfather’s (but not grandmother’s) slow-growth period was associated with an increase in diabetes mortality.^^19^^

In a follow-up study of the same Överkalix individuals, Pembrey and colleagues found further evidence of sex-specific multigenerational effects: Male descendants had a statistically increased relative risk of mortality if the paternal grandfather had a good food supply during his slow-growth period, while females had statistically higher relative risks if the paternal grandmother had good food availability during *her* slow-growth period.^^12^^

Other data come from the grandchildren of women who were pregnant in the Western Netherlands in the winter of 1944–1945, when nutritional intake dropped to as little as 400 calories per day as a result of food import restrictions by the occupying German army. In 2008 researchers led by Roseboom reported that the children of women who were exposed to famine *in utero* tended to be fatter at birth and more prone to health problems in adulthood than the children of women born before or after the famine.^^10^^ In earlier studies, Roseboom and colleagues had reported that adult F_1_ populations exposed to famine conditions *in utero* had higher rates of cardiovascular disease,^^20^^^^,^^^^21^^ diabetes,^^22^^^^,^^^^23^^ obesity,^^24^^ and breast cancer.^^25^^

**Figure 3:**
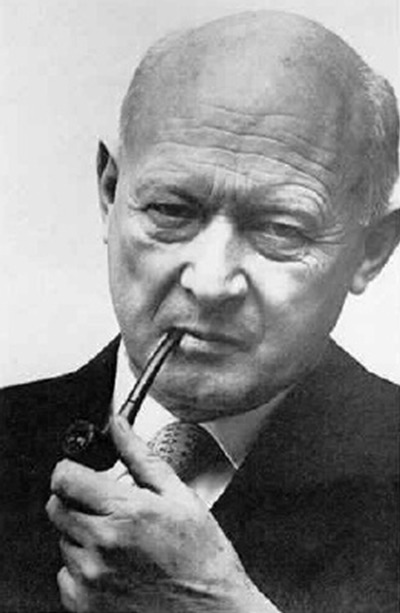
**Epigenetics** is grounded in the work of Conrad Waddington in the 1940s, who coined the term and used it to describe non-Mendelian phenomena influenced by the environment. Many years earlier, French biologist Jean-Baptiste Lamarck had postulated that an organism can pass on traits acquired during its own lifetime. Lamarck’s theories, published 50 years before Darwin’s On the Origin of Species, were accepted by Darwin and others until the rise in the early 1900s of Mendelian genetics, which holds that inherited traits come solely through genes. © The Royal Society (1977)

The F_1_ mothers in the 2008 study completed questionnaires about the birth conditions and current health status of their grown children. The questionnaire grouped health outcomes into four categories: congenital, cardiovascular and metabolic, psychiatric, and other. The only statistically significant association between ancestral famine exposure and poor health outcomes was with the “other” category, which included accidents and acquired neurological, autoimmune, infectious, respiratory, neoplastic, and dermatological conditions. In their conclusions, Roseboom and colleagues state that the findings “constitute the first direct evidence in humans that the detrimental effects of poor maternal nutrition during gestation on health in later life pass down to subsequent generations.”^^10^^

Roseboom calls the findings “a first but weak” indication of multigenerational effects on health after prenatal famine exposure. “It was weak because we approached the F_1_ and not the F_2_ directly,” she explains. “But in a next study we contacted the F_2_ directly, and we found they were more adipose not only at birth but also currently while in their forties, and therefore we expect that they might have increased cardiovascular disease rates later on in their lives.”

Another key line of human evidence in the field comes from multigenerational studies of DES.^^9^^ Those data came from a pair of National Cancer Institute studies: the DES Follow-Up Study, which tracks health outcomes among women who were exposed to DES and their prenatally exposed children, and the DES Third Generation Cohort Study, which tracks the male and female grandchildren of the originally exposed women.

According to Linda Titus, a professor in community and family medicine and pediatrics at the Geisel School of Medicine at Dartmouth, grandsons of DES-exposed women had a modestly higher risk of any birth defect, mostly urogenital defects, although the findings weren’t statistically significant. Granddaughters, meanwhile, had a higher frequency of hip dysplasia, irregular periods, older age at menarche, and potentially an increased risk of infertility. There was also a higher risk of ovarian cancer among granddaughters of exposed women, but since that finding is based on just three cases, she says, it must be considered preliminary.^^26^^

## Epigenetic Evidence in Humans Still Emerging

The human data on epigenetics is generally limited to F_1_ populations and comes mainly from studies on the Dutch famine.^^8^^^,^^^10^^^,^^^11^^^^,^^^^12^^ According to Roseboom, the first study to link undernutrition during gestation to altered epigenetic status was published by Bastian T. Heijmans, an associate professor of genetics at Leiden University Medical Center.^^8^^ In that study, Heijmans and colleagues reported that F_1_ generations exposed to Dutch famine conditions *in utero* had hypomethylation of the *IGF2* gene six decades later, compared with same-sex siblings not exposed to famine (they noted that other stressors such as cold and emotional stress could have contributed to the observed hypomethylation).

According to Roseboom, this finding suggests prenatal famine could lead to changes in gene expression via changes in methylation. But Heijmans’ research team was not able to statistically associate hypomethylated *IGF2* with any specific health outcomes. And Roseboom points out that “whether these changes in methylation actually result in changes in gene expression and ultimately changes in, for instance, cardiovascular risk factors remains to be investigated.”

Roseboom’s team followed up last year with a study investigating four additional genes that have been shown in animals to be persistently altered by maternal dietary restrictions. But the study failed to demonstrate any consistent links between famine exposure and methylation status, possibly because of confounding from lifestyle choices and diet later in life.^^13^^ Roseboom’s team is currently analyzing methylation levels on DNA obtained from the F_0_, F_1_, and F_2_ generations affected by the Dutch famine; these data have not yet been submitted for publication.

Titus says that conclusive evidence of transgenerational epigenetic mechanisms in humans will depend on findings in F_3_ generations. “Even if new studies confirm outcomes in DES-exposed grandchildren, we can’t be sure if they are due to epigenetic changes,” she says. “A true assessment of heritable epigenetic changes requires studies of great-grandchildren, which will be the first generation without DES exposure.”

Blumberg emphasizes that just because the data haven’t yet materialized doesn’t mean that environmentally induced, transgenerational epigenetic changes in humans don’t occur. “We see transgenerational epigenetic changes in animals, and what we believe is that the animal data predict human responses,” he says. “Moreover, it’s possible that you won’t see epigenetic changes from looking at genes—you might see it, instead, in noncoding regions in DNA.”

The growing evidence that environmental exposures might induce a myriad of effects that persist transgenerationally leaves open questions about where human evolution is headed, Crews asserts. “It’s a new window on the ‘nature versus nurture’ debate,” he says. “We’re all combinations of what we inherit and what we’re exposed to in our own lives. And right now you can’t find a human or an animal on the planet without a body burden of endocrine-disrupting chemicals.”
